# [μ_3_-2,2,4,4,6,6-Hexakis(3,5-dimethyl­pyrazol-1-yl)-2λ^5^,4λ^5^,6λ^5^-1,3,5,2,4,6-triaza­triphosphinine]tris­[*cis*-dichloridopalladium(II)]

**DOI:** 10.1107/S1600536808023167

**Published:** 2008-07-31

**Authors:** Sung Yol Yun, Soon W. Lee

**Affiliations:** a Department of Chemistry (BK21), Sungkyunkwan University, Natural Science Campus, Suwon 440-746, Republic of Korea

## Abstract

The title complex, [Pd_3_Cl_6_(C_30_H_42_N_15_P_3_)], possesses *C*_3_ mol­ecular symmetry. The P and N atoms of the cyclo­triphosphazene and the Pd atom are located on the crystallographic mirror plane. Each of the three symmetry-related Pd atoms is coordinated by two chloride ligands and two exocyclic pyrazolyl N atoms, but not by the cyclo­triphosphazene N atoms.

## Related literature

For related literature, see: Chandrasekhar & Nagendran (2001 ▶); Gallicano & Paddock (1982 ▶).
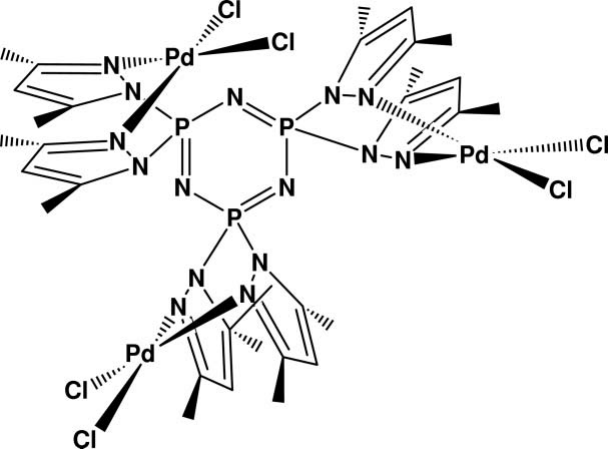


## Experimental

### 

#### Crystal data

[Pd_3_Cl_6_(C_30_H_42_N_15_P_3_)]
*M* *_r_* = 1237.60
Hexagonal, 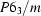

*a* = 17.2989 (3) Å
*c* = 14.4545 (6) Å
*V* = 3746.02 (18) Å^3^
*Z* = 2
Mo *K*α radiation
μ = 1.02 mm^−1^
*T* = 296 (2) K
0.24 × 0.20 × 0.16 mm

#### Data collection

Bruker SMART CCD area-detector diffractometer
Absorption correction: multi-scan (North *et al.*, 1968 ▶) *T*_min_ = 0.792, *T*_max_ = 0.854
42917 measured reflections
3157 independent reflections
2098 reflections with *I* > 2σ(*I*)
*R*_int_ = 0.048

#### Refinement

*R*[*F* ^2^ > 2σ(*F* ^2^)] = 0.035
*wR*(*F* ^2^) = 0.108
*S* = 1.07
3157 reflections
91 parameters
H-atom parameters constrained
Δρ_max_ = 0.52 e Å^−3^
Δρ_min_ = −0.35 e Å^−3^

### 

Data collection: *SMART* (Bruker, 1997 ▶); cell refinement: *SAINT* (Bruker, 1997 ▶); data reduction: *SAINT*; program(s) used to solve structure: *SHELXTL* (Sheldrick, 2008 ▶); program(s) used to refine structure: *SHELXTL*; molecular graphics: *SHELXTL*; software used to prepare material for publication: *SHELXTL*.

## Supplementary Material

Crystal structure: contains datablocks global, I. DOI: 10.1107/S1600536808023167/kp2182sup1.cif

Structure factors: contains datablocks I. DOI: 10.1107/S1600536808023167/kp2182Isup2.hkl

Additional supplementary materials:  crystallographic information; 3D view; checkCIF report

## Figures and Tables

**Table:** 

Pd1—N3	2.027 (2)
Pd1—Cl1	2.2642 (10)
P1—N2	1.695 (2)
N2—N3	1.384 (3)

**Table:** 

N3—Pd1—N3^i^	86.36 (14)
N3—Pd1—Cl1	178.26 (8)
N3^i^—Pd1—Cl1	92.34 (8)
Cl1—Pd1—Cl1^i^	88.95 (6)
N1^ii^—P1—N1	118.0 (2)
N2^i^—P1—N2	102.86 (17)

Symmetry codes: (i) 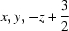; (ii) 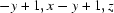.

**Fig. 1.**
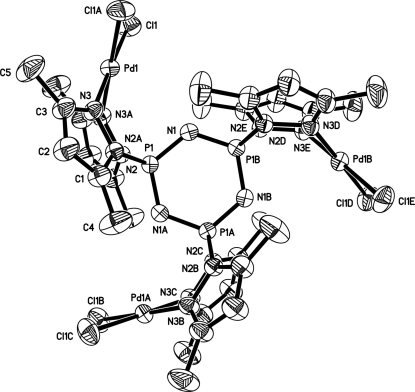
Molecular structure showing the 50% probability displacement ellipsoids. H atoms are omitted for clarity.

**Fig. 2.**
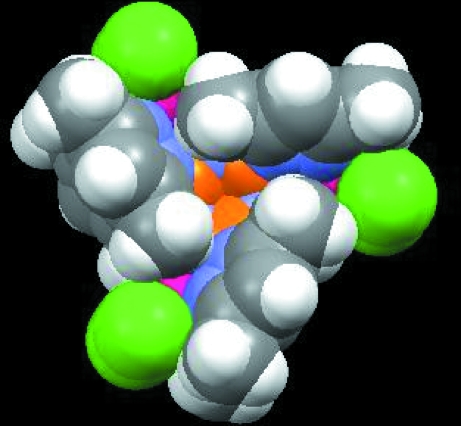
A space filling model of the title complex showing its C3 axis at the center of the cyclotriphosphazene ring: (a) red: Pd; green: Cl; orange: P; purple: N; grey: C; white, H.

## supplementary crystallographic information

### Comment

Various cyclotriphosphazene-based ligands have been designed and utilized to prepare coordination and organometallic complexes (Chandrasekhar & Nagendran, 2001). In particular, the 6-membered cyclic ligand N_3_P_3_(3,5-Me_2_pz)_6_ (3,5-Me_2_pz = 3,5-dimethylpyrazolyl) has many potential donor sites due to the exocyclic pyrazolyl nitrogen atoms in addition to the ring nitrogen and phosphorus atoms. This ligand was previously reported to react with [PdCl_2_(PhCN)_2_] to give the title complex, which was not structurally characterized by X-ray difraction (Gallicano & Paddock, 1982). We chose the title complex to be used as a starting material with the C_3_-symmetry in preparing coordination polymers by treating it with organic linking ligands. In this context, we determined the three-dimensional structure of the title complex to confirm its molecular symmetry.

The central core has a perfectly planar hexagonal P_3_N_3_ unit, to which three surrounding square-planar palladium fragments (PdCl_2_N_2_) are perpendicular (Fig. 1, Table 1). The crystallographic mirror plane (*z* = 3/4) passes through the central cyclotriphosphazene ring (three P and three N atoms) and the three surrounding palladium atoms, and bisects the pendant germinal pyrazolyl ligands in each, symmetry related PdCl_2_(pyrazolyl)_2_ unit. A space filling model of the title complex (Fig. 2) shows its C_3_-symmetry and close packing. As previously predicted by  NMR and IR spectroscopy (Gallicano & Paddock, 1982), each palladium metal is coordinated by two chloro ligands and exocyclic pyrazolyl N atoms, but not to the cyclotriphosphazene N atoms, and lies 0.022 (1) Å below the Cl_2_N_2_ plane. Each phosphorus atom is bound to four N atoms: two central cyclotriphosphazene N atoms and two exocyclic pyrazolyl N atoms. Consistently with our expectation, the P1—N1 (cyclotriphosphazene) bond is significantly longer than P1—N2 (pyrazolyl) bond. All the Pd···Pd separations are equal (7.7538 (6) Å) due to the crystallographic symmetry.

### Experimental

The title complex was prepared by the literature method (Gallicano & Paddock, 1982). The product was recrystallized from a mixture of dichloromethane–hexane.

### Refinement

All non-hydrogen atoms were refined anisotropically. All hydrogen atoms were generated in ideal positions and refined in a riding model.

### Crystal data

**Table:**

[Pd_3_Cl_6_(C_30_H_42_N_15_P_3_)]	*Z* = 2
*M**_r_* = 1237.60	*F*_000_ = 1224
Hexagonal, *P*6_3_/*m*	*D*_x_ = 1.097 Mg m^−^^3^
Hall symbol: -P6c	Mo *K*α radiation λ = 0.71073 Å
*a* = 17.2989 (3) Å	Cell parameters from 9849 reflections
*b* = 17.2989 (3) Å	θ = 2.4–27.2º
*c* = 14.4545 (6) Å	µ = 1.02 mm^−^^1^
α = 90º	*T* = 296 (2) K
β = 90º	Block, yellow
γ = 120º	0.24 × 0.20 × 0.16 mm
*V* = 3746.02 (18) Å^3^	

### Data collection

**Table:**

Bruker SMART CCD area-detector diffractometer	3157 independent reflections
Radiation source: sealed tube	2098 reflections with *I* > 2σ(*I*)
Monochromator: graphite	*R*_int_ = 0.048
*T* = 296(2) K	θ_max_ = 28.3º
φ and ω scans	θ_min_ = 3.6º
Absorption correction: multi-scan(North *et al.*, 1968)	*h* = −23→22
*T*_min_ = 0.792, *T*_max_ = 0.854	*k* = −19→22
42917 measured reflections	*l* = −19→19

### Refinement

**Table:**

Refinement on *F*^2^	Secondary atom site location: difference Fourier map
Least-squares matrix: full	Hydrogen site location: inferred from neighbouring sites
*R*[*F*^2^ > 2σ(*F*^2^)] = 0.035	H-atom parameters constrained
*wR*(*F*^2^) = 0.108	*w* = 1/[σ^2^(*F*_o_^2^) + (0.0477*P*)^2^ + 1.7582*P*] where *P* = (*F*_o_^2^ + 2*F*_c_^2^)/3
*S* = 1.07	(Δ/σ)_max_ = 0.002
3157 reflections	Δρ_max_ = 0.52 e Å^−^^3^
91 parameters	Δρ_min_ = −0.35 e Å^−^^3^
Primary atom site location: structure-invariant direct methods	Extinction correction: none

### Special details

**Table:**

Geometry. All e.s.d.'s (except the e.s.d. in the dihedral angle between two l.s. planes) are estimated using the full covariance matrix. The cell e.s.d.'s are taken into account individually in the estimation of e.s.d.'s in distances, angles and torsion angles; correlations between e.s.d.'s in cell parameters are only used when they are defined by crystal symmetry. An approximate (isotropic) treatment of cell e.s.d.'s is used for estimating e.s.d.'s involving l.s. planes.
Refinement. Refinement of *F*^2^ against ALL reflections. The weighted *R*-factor *wR* and goodness of fit *S* are based on *F*^2^, conventional *R*-factors *R* are based on *F*, with *F* set to zero for negative *F*^2^. The threshold expression of *F*^2^ > σ(*F*^2^) is used only for calculating *R*-factors(gt) *etc*. and is not relevant to the choice of reflections for refinement. *R*-factors based on *F*^2^ are statistically about twice as large as those based on *F*, and *R*- factors based on ALL data will be even larger.

### Fractional atomic coordinates and isotropic or equivalent isotropic displacement parameters (Å^2^)

**Table:**

	*x*	*y*	*z*	*U*_iso_*/*U*_eq_	
Pd1	0.13572 (2)	0.37375 (2)	0.7500	0.05476 (14)	
Cl1	0.02844 (7)	0.32969 (8)	0.64026 (8)	0.0961 (3)	
P1	0.31769 (7)	0.56802 (7)	0.7500	0.0438 (2)	
N1	0.2362 (2)	0.5861 (2)	0.7500	0.0472 (7)	
N2	0.30675 (15)	0.50221 (15)	0.84168 (15)	0.0503 (5)	
C1	0.3607 (2)	0.5175 (2)	0.9179 (2)	0.0697 (9)	
C2	0.3213 (3)	0.4397 (3)	0.9672 (3)	0.0873 (12)	
H2	0.3425	0.4290	1.0220	0.105*	
N3	0.23411 (17)	0.41658 (16)	0.84595 (17)	0.0573 (6)	
C3	0.2439 (2)	0.3792 (2)	0.9210 (3)	0.0743 (10)	
C4	0.4433 (3)	0.6016 (3)	0.9398 (3)	0.1093 (17)	
H4A	0.4555	0.6442	0.8914	0.164*	
H4B	0.4921	0.5904	0.9449	0.164*	
H4C	0.4359	0.6249	0.9974	0.164*	
C5	0.1818 (3)	0.2838 (3)	0.9456 (4)	0.121 (2)	
H5A	0.1345	0.2576	0.9008	0.181*	
H5B	0.1570	0.2805	1.0058	0.181*	
H5C	0.2139	0.2518	0.9458	0.181*	

### Atomic displacement parameters (Å^2^)

**Table:**

	*U*^11^	*U*^22^	*U*^33^	*U*^12^	*U*^13^	*U*^23^
Pd1	0.0519 (2)	0.0567 (2)	0.05085 (19)	0.02349 (16)	0.000	0.000
Cl1	0.0739 (6)	0.1049 (8)	0.0898 (7)	0.0300 (6)	−0.0295 (5)	−0.0085 (6)
P1	0.0508 (6)	0.0488 (6)	0.0333 (4)	0.0259 (5)	0.000	0.000
N1	0.0495 (18)	0.0505 (19)	0.0375 (15)	0.0218 (15)	0.000	0.000
N2	0.0538 (14)	0.0508 (13)	0.0433 (11)	0.0240 (12)	−0.0024 (10)	0.0062 (9)
C1	0.071 (2)	0.072 (2)	0.0544 (17)	0.0276 (18)	−0.0132 (15)	0.0103 (15)
C2	0.088 (3)	0.080 (2)	0.073 (2)	0.026 (2)	−0.019 (2)	0.0274 (19)
N3	0.0636 (16)	0.0503 (14)	0.0518 (13)	0.0238 (12)	−0.0023 (11)	0.0097 (10)
C3	0.074 (2)	0.064 (2)	0.068 (2)	0.0219 (18)	−0.0088 (17)	0.0191 (16)
C4	0.101 (3)	0.095 (3)	0.072 (2)	0.005 (2)	−0.041 (2)	0.024 (2)
C5	0.117 (4)	0.079 (3)	0.122 (4)	0.016 (3)	−0.028 (3)	0.047 (3)

### Geometric parameters (Å, °)

**Table:**

Pd1—N3	2.027 (2)	C1—C4	1.476 (5)
Pd1—N3^i^	2.027 (2)	C2—C3	1.389 (5)
Pd1—Cl1	2.2642 (10)	C2—H2	0.9300
Pd1—Cl1^i^	2.2641 (10)	N3—C3	1.317 (4)
P1—N1^ii^	1.557 (3)	C3—C5	1.494 (5)
P1—N1	1.589 (3)	C4—H4A	0.9600
P1—N2^i^	1.695 (2)	C4—H4B	0.9600
P1—N2	1.695 (2)	C4—H4C	0.9600
N1—P1^iii^	1.557 (3)	C5—H5A	0.9600
N2—C1	1.381 (4)	C5—H5B	0.9600
N2—N3	1.384 (3)	C5—H5C	0.9600
C1—C2	1.367 (5)		
			
N3—Pd1—N3^i^	86.36 (14)	C1—C2—H2	126.0
N3—Pd1—Cl1	178.26 (8)	C3—C2—H2	126.0
N3^i^—Pd1—Cl1	92.34 (8)	C3—N3—N2	107.0 (2)
N3—Pd1—Cl1^i^	92.34 (8)	C3—N3—Pd1	132.5 (2)
N3^i^—Pd1—Cl1^i^	178.26 (8)	N2—N3—Pd1	120.51 (16)
Cl1—Pd1—Cl1^i^	88.95 (6)	N3—C3—C2	109.7 (3)
N1^ii^—P1—N1	118.0 (2)	N3—C3—C5	122.7 (3)
N1^ii^—P1—N2^i^	108.78 (11)	C2—C3—C5	127.4 (3)
N1—P1—N2^i^	108.66 (11)	C1—C4—H4A	109.5
N1^ii^—P1—N2	108.78 (11)	C1—C4—H4B	109.5
N1—P1—N2	108.66 (11)	H4A—C4—H4B	109.5
N2^i^—P1—N2	102.86 (17)	C1—C4—H4C	109.5
P1^iii^—N1—P1	122.0 (2)	H4A—C4—H4C	109.5
C1—N2—N3	109.5 (2)	H4B—C4—H4C	109.5
C1—N2—P1	131.1 (2)	C3—C5—H5A	109.5
N3—N2—P1	119.37 (17)	C3—C5—H5B	109.5
C2—C1—N2	105.7 (3)	H5A—C5—H5B	109.5
C2—C1—C4	128.3 (3)	C3—C5—H5C	109.5
N2—C1—C4	126.0 (3)	H5A—C5—H5C	109.5
C1—C2—C3	108.1 (3)	H5B—C5—H5C	109.5

Symmetry codes: (i) *x*, *y*, −*z*+3/2; (ii) −*y*+1, *x*−*y*+1, *z*; (iii) −*x*+*y*, −*x*+1, *z*.
